# Consolidating evidence on the role of insulin resistance in major depressive disorder

**DOI:** 10.1097/YCO.0000000000000905

**Published:** 2023-11-09

**Authors:** Anna Julia Krupa, Dominika Dudek, Marcin Siwek

**Affiliations:** a Department of Affective Disorders; b Department of Adult Psychiatry, Jagiellonian University Collegium Medicum, Krakow, Poland

**Keywords:** antidepressants, atypical depression, insulin resistance, major depressive disorder, personalized depression treatment

## Abstract

**Purpose of review:**

The circular interactions between type 2 diabetes (TMD2) and major depressive disorder (MDD) are well documented but the understanding of their mechanisms has only recently gained more clarity. Latest research indicates, that the association between TMD2 and MDD is largely mediated by insulin resistance (IR).

**Recent findings:**

A metabolic subtype of MDD can be distinguished from other MDD subpopulations, that is characterized by predominantly atypical clinical presentation, IR and different responsiveness to antidepressant interventions. IR is a predictor of nonresponse to some antidepressants. The IR seems to be a state-marker of clinical or subclinical depression and the relationship between IR and MDD varies between sexes and ethnicities. Insulin has a direct impact on the monoaminergic systems known to underlie MDD symptoms: serotoninergic and dopaminergic, which are dysregulated in IR subjects. Several trials assessed the efficacy of insulin-sensitizing drugs in MDD with mixed results for metformin and more consistent evidence for pioglitazone and lifestyle intervention/physical activity.

**Summary:**

Recently published data suggest a significant role of IR in the clinical presentation, pathophysiology and treatment response in MDD. Further research of IR in MDD and integration of existing data into clinical practice are needed.

## INTRODUCTION

The bidirectional link between type 2 diabetes (TMD2) and depression has long been documented, however the precise mechanisms underlying this association remained elusive and so did their importance to clinical care for individuals with major depressive disorder (MDD) [1]. Given that MDD is one of the leading causes of disability worldwide and its impact is expected to rise, it is of key importance to develop its understanding and management [2]. Conversely, TMD2 is too among most important causes of disability worldwide and is highly comorbid with MDD, with one disorder increasing the risk of another twofold. A latter-day study in Italian families burdened with TMD2 and MDD indicated that CRHR2 gene pleiotropism might underlie the diabetes and depression comorbidity [3]. TMD2 exacerbates MDD, while MDD is associated with more complications, higher disability and mortality in TMD2 [4]. It was also reported that the relationship between TMD2 and mood disturbance is largely mediated by insulin resistance (IR) [5]. Although less studies explored the relationship between IR and MDD without TMD2, it was noted that IR is linked to higher severity of depressive symptoms in the general population [6^▪▪^] and that, compared to non-MDD individuals, MDD subjects present higher IR and IR is linked to higher MDD morbidity [7]. Furthermore, in a systematic review it was reported that treatment of MDD with insulin-sensitizing drugs significantly improved depression. Interestingly, the majority of these studies did not confirm a correlation between improvement of metabolic parameters and depression symptomatology, suggesting other potential mechanisms of their antidepressant efficacy. These data must be interpreted with caution, as the studies were based on small, heterogenous groups (with: no metabolic comorbidities/metabolic syndrome/insulin resistance/TMD2) and various measures of metabolic parameters were applied [8], nonetheless they warrant further investigation. The aim of this narrative review is to summarize the latest literature on the role of insulin resistance in MDD. 

**Box 1:**
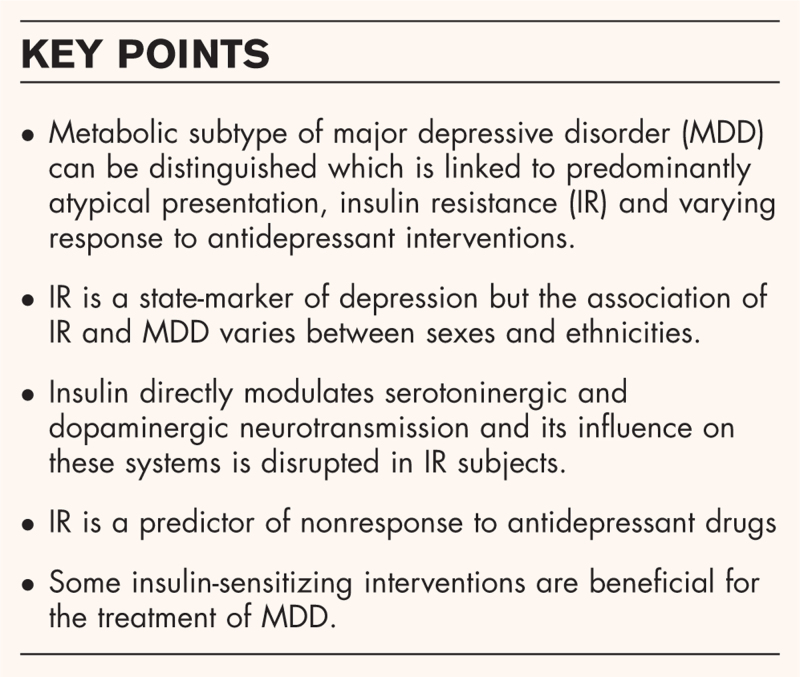
no caption available

## METABOLIC SUBTYPE OF DEPRESSION

Hence the high heterogeneity of MDD, several authors attempted to distinguish more homogenous subtypes of MDD and link them to particular biomarkers. Brouwer *et al.*[9] examined MDD patients with comorbid TMD2, using the hyperinsulinemic euglycemic clamp (HEC) procedure, and explored the association of IR to MDD clinical presentation and response to light therapy. Participants were selected to higher/lower IR groups based on the M-value (measure of whole-body IR based on the amount of glucose infusion during HEC). Subjects with higher IR varied in depressive symptomatology from those with lower IR, with the former group presenting more pronounced irritability, anhedonia, fatigue and hypersomnia and the latter group demonstrating higher severity of insomnia. The results are in congruence with a large meta-analysis by Fernandes *et al.*[10^▪▪^], which included both cross-sectional and longitudinal studies. Their work showed, that IR was elevated in atypical but not typical depression. Some researchers focused on specific symptoms and attempted to disentangle their links to metabolic markers. Chae *et al.*[11^▪▪^] reported an analysis of German nationwide database which assessed data on the clinical presentation, metabolic syndrome components and inflammation variables in MDD patients. Regarding insulin levels, results showed significant associations between higher insulin and increased appetite, hypersomnia, insomnia and suicidal ideation. In sum their work unraveled several symptom-marker connections between: higher metabolic markers and increased appetite; lower metabolic markers and decreased appetite; lower metabolic markers and insomnia; higher insulin and increased appetite; higher insulin and lower albumin and insomnia. In line with the findings of the Netherlands Study of Depression and Anxiety [12^▪▪^], their work reinforced the links between metabolic markers and atypical presentation of MDD and the observation, that those relationships are mainly noted for insomnia rather than hypersomnia. Similarly, in a cross-sectional analysis of primary care population Shell *et al.*[13^▪^] detected significant links between IR and increased food intake, hypersomnia (positive) and reduced food intake (negative). The relationship between IR and overeating was only significant for White participants but not for Black participants. This study also indicated that the associations between IR and overeating as well as hypersomnia were partially mediated by BMI. Of note, Łapińska *et al.*[14] observed that IR was also linked to subclinical depression symptoms assessed with the somatic-vegetative subscale of Beck Depression Inventory (BDI) (but not cognitive-affective items). Furthermore, in a cross-sectional study of Chinese MDD subjects it was noted, that IR was associated with increased likelihood of suicide attempts [15]. What is more, a cross-sectional study in middle-aged subjects showed, that higher IR was not only a predictor of higher depressive symptoms but also worse executive functions [16^▪^].

## STATE OR TRAIT

The above-mentioned meta-analysis by Fernandes *et al.*[10^▪▪^] reported that IR is only elevated during acute MDD episodes, while comparisons of patients with remitted MDD and general population indicated no significant differences. Another work comparing subjects with current MDD, remitted MDD and controls also concluded, that IR was significantly higher in acute MDD vs. controls [17]. Rashidian *et al.*[18^▪▪^] noted that IR decreased in patients who experienced symptomatic improvement. They also noted that while baseline C-reactive protein (CRP) levels had a significant effect on the changes in depression severity it was fully mediated by IR. A study comparing IR in normal weight, nondiabetic adolescents included MDD and euthymic participants. It found associations between MDD and IR, which became insignificant after controlling for age, sex and BMI. However, the study sample was small (*n* = 196) and less than a fourth of subjects presented IR (defined as HOMA >2.5), therefore the study was potentially underpowered to detect IR related differences [19]. Moreover, a study in overweight/obese patients without clinical MDD or TMD2 found that subclinical depressive symptoms are linked to IR. Of note, while the association between IR and subclinical depression was moderated by family history of TDM2, it was completely independent of BMI [20].

## SEX DIFFERENCES

He *et al.*[21^▪▪^] published a cross-sectional study exploring the links between IR and depression status in obese individuals without diabetes, not medicated with antidiabetic drugs. They observed, that in female subjects IR (defined as 4th quartile vs. 1st–3rd quartiles HOMA that is >5.5) was significantly associated with mild, moderate or severe depression, with higher depression severity being related to higher risk of IR. To the contrary, no significant link between IR and depression was noted in male participants. Łapińska *et al.*[14] studied a mixed group of women with normal glucose tolerance prediabetes and diabetes, additionally performing the analyses in postmenopausal subjects to explore the relationship between IR and subclinical depression (individuals with clinical depression were excluded). It was reported that IR was higher in women with subclinical depressive symptoms compared to those in euthymia, both in the whole group and in the postmenopausal subset. Zheng *et al.*[22^▪▪^] performed a longitudinal assessment of links between baseline IR and depressive symptoms measured biennially for 8 years. The results indicated that high IR (4th vs. 1st quartile of studied population) was associated with progression of depressive symptoms. This relationship was significant in subgroups of men, overweight and elderly subjects but not in women, participants with normal BMI, those aged <65 years.

## NEUROTRANSIMSSION

The alterations of serotonin, dopamine transmission are known to induce depressive symptoms. In an animal study, Martin *et al.* discovered that insulin (i) directly inhibits the activity of serotoninergic neurons in the dorsal raphe nucleus (DRN) via 5-HT1A autoreceptor dependent mechanism, (ii) exerts its anxiolytic-like effects due to modulation of serotoninergic transmission, (iii) in animal models of T2D-related depression serotoninergic neurons in DRN are no longer sensitive to insulin, (iv) in animal models of depression addition of insulin to fluoxetine potentiated the antidepressant action of the drug [23^▪▪^]. On the other hand, Gruber *et al.*[24^▪▪^] summarized data coming from animal and human trials exploring the impact of insulin and IR on dopaminergic transmission. Based on the animal studies they have concluded that (a) insulin differently modulates dopamine release, increasing it if applied in the striatum and inhibiting it if applied in the ventral tegmental area (VTA), (b) insulin deficiency and resistance limits the synthesis and reuptake of dopamine as well as the excitatory stimulation and firing frequency of dopaminergic neurons in VTA, (c) restricted insulin signaling/IR in the nucleus accumbens reduces dopamine release and reuptake, (d) IR is linked to increased food intake, motivation to work for food, dampens reward sensitivity, impairs learning and preference formation as well as promotes behavioral despair resulting in anhedonic/anxious phenotype. Moreover, they summed human studies noting that (i) peripheral IR is a surrogate measure of central IR, (ii) intranasal insulin alters the activity and connectivity of dopaminergic circuitry and modulates reward behavior, (iii) the effects of intranasal insulin on reward processing differs with its dose, with low and high doses increasing the thresholds for reward the most, (iv) IR disrupts the reactivity to food cues, learning and motivation for reward (the last association is highly related to the state of satiety/hunger). While the relationship between high BMI and dysregulation of reward processing of food cues and intake is well established, a study exploring the effects of intranasal insulin on functional connectivity of the dopaminergic midbrain in humans reported, that the, these alterations are better predicted by IR rather than BMI [25].

## ANTIDEPRESSANT TREATMENT EFFICACY

Surprisingly, IR seems to differently moderate treatment outcomes depending on its modality. In the earlier mentioned work by Brouwer *et al.*[9], the treatment with light therapy in patients with higher IR significant improvement was noted in the domains of interpersonal sensitivity, self-criticism, guilt, psychomotor agitation, concentration/decision making and appetite, while to the contrary, in the lower IR subgroup it improved interest in people/activities and morning awakening but exacerbated sadness, irritability as well as interpersonal sensitivity. When analyzing posthoc a prospective study the links between IR and response to vortioxetine in MDD, Rashidian *et al.*[18^▪▪^,^26^] observed that (i) IR was a negative predictor of treatment response to vortioxetine, (ii) IR predicted decreased improvement in anhedonia early in the treatment and worse self-rated cognitive and psychosocial functioning after 8 weeks of treatment, (iii) IR decreased in subjects who achieved treatment response while it increased in those who did not. In a study of fibromyalgia patients, of which 58% were comorbid with MDD, we found that IR, depression, anxiety and personality disorders were predictors of nonresponse to serotonin and noradrenaline reuptake inhibitors. Of interest, while subjects resistant to SNRI had significantly higher BMI than those responsive to SNRI, the relationship between BMI and resistance to SNRI was fully mediated by IR [27^▪^]. Also, we recently presented a pilot analysis exploring the links between IR and response to treatment with SNRI in MDD. The results indicated that MDD subjects resistant to SNRI were characterized by higher IR and BMI than those responsive to SNRI [28]. On the other hand, trials exploring the effectiveness of psychotherapy in adolescent girls with depression at risk of TMD2 failed to show the link between improvement of depression and IR [29].

## INSULIN-SENSITIZING DRUGS (OFF-LABEL)/INTERVENTIONS IN MAJOR DEPRESSIVE DISORDER TREATMENT

Moulton *et al.*[30] performed a systematic review and meta-analysis of antidiabetic treatments effects on depressive symptoms. Eight randomized controlled studies (RCTs) and two open-label trials were available for pioglitazone treatment. In the majority of these studies patients were diagnosed with MDD in the course of bipolar disorder/Parkinson's Disease/post-stroke/with history of alcohol use disorder rather than unipolar MDD alone and received mood stabilizers/antipsychotics/antidepressants, the groups were heterogeneous regarding the metabolic characteristics (TMD2/metabolic syndrome/insulin resistance/nondiabetic). They concluded that pioglitazone vs. placebo effectively reduced severity of depression and was more effective than metformin in alleviating depression. Interestingly, reduction in depressive symptoms was linked to female sex, but not to baseline IR or severity of depression. One observation reported positive effect of rosiglitazone on depression symptoms. Six RCTs were available for metformin. Their analysis suggested that the effect of metformin on depression severity was comparable to placebo, but they noted a high heterogeneity among studies. In one RCT, liraglutide was comparable to placebo in patients with TMD2, while in an open observation of subjects with depression and cognitive impairment liraglutide significantly alleviated depression. In a group of patients with poorly controlled TMD2 insulin treatment did not improve depression. More recently, Calkin *et al.*[31] published results of a RCT assessing the effects of add-on metformin vs. placebo in patients with treatment resistant bipolar depression (TRBD) and IR. They observed a significantly higher percentage of subjects achieving the normalization of insulin sensitivity after 14 weeks of treatment in metformin group vs. placebo group (55% vs. 4%). In participants with normalized insulin sensitivity vs. with IR significant improvements in the severity of depression, anxiety as well as general functioning were noted. Reportedly, further studies by these researchers assessing the effect of semaglutide and pioglitazone in TRBD are underway. What is more, it has been reported that intranasal insulin vs. placebo increased positive affect, food reward and reduced snacking in women and that this effect was more pronounced in obese but not lean participants [32]. Luciano *et al.*[33^▪^] reported a prospective multisite study in which patients with schizophrenia, bipolar disorder and MDD were allocated to a 6-month group intervention promoting healthy lifestyle behaviors or brief psychoeducational group intervention as a control. Metabolic parameters [BMI, weight, waist circumference, Cumulative Illness Rating Scale (CIRS), lipid profile, Framingham risk score, HOMA] were assessed at baseline, after 6 and 12 months. It was noted that in the MDD subgroup metabolic parameters (BMI, weight, waist circumference, fasting glucose, IR, lipid profile) and symptom severity in domains of affectivity, negative symptoms, activity as well as quality of life significantly improved with the lifestyle intervention vs. control. Donyaei *et al.*[34^▪^] assessed the effectiveness of 12-week aerobic-resistance training program on the metabolic parameters, BDNF and severity of depression in TMD2. Initial BDI indicated that both control and experimental groups showed severe levels of depression. Results indicated significant improvement in BDI and IR. After 8 weeks of detraining, both IR and depression levels increased again, yet IR remained significantly lower vs. baseline while depression severity was comparable to baseline.

## CONCLUSION

In sum, latest research suggests that a metabolic subtype of MDD can be distinguished from other MDD populations by the presence of particular pathophysiological mechanisms, symptomatology and responsiveness to treatment. IR seems to play an important role in this metabolic MDD subtype and it is positively linked to atypical depressive symptomatology. Insulin has a direct effect on the serotoninergic and dopaminergic neurotransmission which becomes disrupted by IR. The relationship between IR and MDD seems restricted to current depressive states of either clinical of subclinical severity and it differs between sexes, ethnicities normal/overweight subjects. Moreover, IR limits the efficacy of antidepressant drugs but light therapy might be more effective for subjects with IR than insulin sensitive ones. Insulin-sensitizing treatments might be beneficial for MDD patients, with most robust data supporting the addition of pioglitazone or interventions promoting healthy lifestyle and physical activity. Studies exploring the role of IR in MDD need replication in larger groups/patients of different ethnicities/in various metabolic states (normal/IR/TMD2) and so do trials verifying the potential efficacy of insulin-sensitizing treatments in MDD. Existing data already allow for a step towards personalizing therapeutic approaches and may inform clinical decisions as well as future research for more individualized and efficacious MDD treatment.

## Acknowledgements

*None.*

### Financial support and sponsorship

*None.*

### Conflicts of interest

*There are no conflicts of interest.*

## REFERENCES AND RECOMMENDED READING

Papers of particular interest, published within the annual period of review, have been highlighted as:

▪ of special interest
▪▪ of outstanding interest
